# Maternal Immunization Against Respiratory Syncytial Virus: An Integrative Review of Efficacy, Safety, and Implementation

**DOI:** 10.3390/vaccines14070624

**Published:** 2026-07-16

**Authors:** Isadora Rodrigues Almeida, Marcela Fermoselle de Vita Silva, Taline de Brito Cavalcante, Giovanna Alves de Britto, Manuela Cândido Amaral dos Reis, Luis Fernando Lima Bueno, Luana dos Santos Ribeiro, Gustavo Yano Callado, Susana Cristina Aidé Viviani Fialho, Antonio Braga, Glória Calagna, Edward Araujo Júnior

**Affiliations:** 1 Discipline of Woman Health, Municipal University of São Caetano do Sul (USCS), São Caetano do Sul 09521-160, SP, Brazil; isadora.almeida1@uscsonline.com.br (I.R.A.); marcela.silva1@uscsonline.com.br (M.F.d.V.S.); taline.cavalcante@uscsonline.com.br (T.d.B.C.); giovanna.britto@uscsonline.com.br (G.A.d.B.); manuela.reis@uscsonline.com.br (M.C.A.d.R.); luana.ribeiro@uscsonline.com.br (L.d.S.R.); araujojred@terra.com.br (E.A.J.); 2 Discipline of Gynecology and Obstetrics, Santo Amaro University (UNISA), São Paulo 04829-300, SP, Brazil; luisfernandolimabuenooficial@gmail.com; 3 Albert Einstein Israelite College of Health Sciences (FICSAE), Albert Einstein Israelite Hospital, São Paulo 05652-900, SP, Brazil; gycallado@gmail.com; 4 Department of Maternal and Child Health, School of Medicine, Fluminense Federal University (UFF), Niterói 24070-090, RJ, Brazil; susanaaide@id.uff.br; 5 Department of Gynecology and Obstetrics, School of Medicine, Federal University of Rio de Janeiro (UFRJ), Rio de Janeiro 22240-003, RJ, Brazil; bragamed@yahoo.com.br; 6 Postgraduate Program in Applied Health Sciences, University of Vassouras (Univassouras), Vassouras 27700-000, RJ, Brazil; 7 Department of General and Specialized Surgery, School of Medicine and Surgery, Federal University of the State of Rio de Janeiro (UNIRIO), Rio de Janeiro 22290-240, RJ, Brazil; 8 Villa Sofia Cervello Hospital, University of Palermo, 90100 Palermo, Italy; 9 Department of Obstetrics, Paulista School of Medicine—Federal University of São Paulo (EPM-UNIFESP), São Paulo 04023-062, SP, Brazil

**Keywords:** respiratory syncytial virus, maternal immunization, RSVpreF, pregnancy, passive immunity, infant

## Abstract

Respiratory syncytial virus (RSV) is a leading cause of severe lower respiratory tract infection in young infants and accounts for substantial morbidity, hospitalization, and mortality worldwide, with the highest burden concentrated in the first months of life, when immunological and anatomical immaturity favors severe disease. Maternal vaccination has emerged as a strategy to bridge this window of vulnerability through the transplacental transfer of neutralizing antibodies. This integrative review synthesizes scientific articles published between 2022 and 2026, including systematic reviews, meta-analyses, narrative reviews, and position statements from national and international scientific societies, addressing the epidemiology, clinical manifestations, diagnosis, mechanism of action, efficacy, safety, and public health impact of maternal RSV immunization. The bivalent prefusion F protein vaccine (RSVpreF; Abrysvo^®^), administered as a single intramuscular dose between 32 and 36 weeks of gestation, induces maternal IgG that is transferred to the fetus and confers passive protection during the first six months of life. In the phase 3 MATISSE trial, vaccination demonstrated 81.8% efficacy against severe RSV lower respiratory tract infection within 90 days of birth and 69.4% within 180 days, alongside reductions in hospitalization. The safety profile was favorable, with predominantly mild and transient adverse events; a numerical imbalance in preterm birth remains under surveillance and underlies the precautionary gestational window. Maternal vaccination and long-acting monoclonal antibodies (nirsevimab) are best regarded as complementary rather than competing strategies. Current evidence supports maternal immunization as one of the most effective measures to reduce the burden of RSV disease in early infancy.

## 1. Introduction

Respiratory syncytial virus (RSV) is a pathogen of the family *Pneumoviridae*, genus Orthopneumovirus [1]. It is an enveloped, single-stranded RNA virus that has 10 genes and encodes 11 proteins required for its replication and virulence. RSV infections are seasonal outside the tropics and occur mainly in late autumn and winter; they can range from mild forms to pneumonia and bronchiolitis, and their clinical importance is justified primarily in childhood [2].

The microorganism was first recorded in a population of chimpanzees at the Walter Reed Army Institute of Research. The infected apes developed only coryza; however, the handlers who had contact with the animals presented nasal congestion, coryza, malaise, cough, and several days of fever, indicating transmission from the monkeys to humans. The infected individuals showed an immune response specific to the virus, which at that time was called Chimpanzee Coryza Agent (CCA) and would later be recognized as RSV. In 1957, Dr. Robert Chanock, Bernard Roizman, and Ruth Meyers, at the Johns Hopkins Institute, isolated the virus from affected children who had been diagnosed with pneumonia and proved that it was identical to CCA [3].

Before the isolation of the virus, lower respiratory tract infections in children were attributed to influenza virus and parainfluenza; however, it is now known that RSV bronchiolitis is the most frequent LRTI and the leading cause of hospitalization in young infants [4]. Although RSV infection can affect individuals of all age groups, its greatest clinical relevance lies in the first months of life. It is associated with high infant morbidity and mortality, especially in underdeveloped countries and low- and middle-income regions. The Houston Family Study followed 125 infants from 1975 to 1980 and concluded that 68% of the cohort had at least one RSV infection in the first year of life, later influencing the entire cohort, with 95% of individuals affected in the second year of life. However, it was identified that one of the predisposing factors for the severity of an epidemic of the virus is the virulence of the strain circulating in the year in question, with milder variants tending to cause fewer infections and to have a better prognosis [2]. Even so, in 2015, globally, 33.1 million children under 5 years of age had RSV infectious episodes, with 3.2 million hospitalizations and approximately 60,000 in-hospital deaths; in the community, this figure was approximately 118,000. In 2019, an estimate carried out across 58 countries found between 2.5 and 4.1 million hospitalizations in children under 5 years of age, although this figure is believed to be underestimated owing to the difficulty in recording deaths, especially in the community, and to underreporting in healthcare facilities in several countries [5].

The greater susceptibility of young infants to severe RSV infection is related to the immaturity of the immune system and to anatomy at this age. In the first months of life, both innate and adaptive immunity have less efficient responses, since they have a lower capacity to recognize and respond to pathogen aggressions. The newborn has reduced amounts of cytokines, particularly interferons, which are needed to halt viral replication. This favors the viral cycle occurring in the airways and increases the risk of lower tract infection. In addition, the lungs complete its maturation after birth, and because it has smaller-caliber structures, even small amounts of edema and inflammation can represent major obstruction. Thus, microbial invasion at this age can compromise gas exchange and lead to severe respiratory failure [6].

Given the prevalence of the disease and the vulnerability of the population involved, the development of tools that can reduce infection rates and improve the morbidity of affected patients becomes a public health issue. In view of the inadequate intrinsic immune response in the most affected population for combating the pathogen, the discussion has today turned to the use of neutralizing antibodies, with maternal vaccination being one of the strategies. The objective of vaccinating the pregnant woman is to increase the amount of these antibodies in her during the 2nd or 3rd trimester of pregnancy, resulting in the vertical transmission of immunity to the infant. Studies emphasize that newborns are seropositive for the antibodies for at least 2.5 months after birth, and during this period the titers decline [7].

Although several high-quality reviews on maternal RSV immunization have recently been published, most have focused primarily on vaccine efficacy and safety. The present integrative review expands upon previous literature by incorporating recently published real-world effectiveness data, addressing implementation challenges and regulatory considerations across different healthcare settings, and providing a detailed comparison between maternal vaccination and infant passive immunization with long-acting monoclonal antibodies. By integrating evidence from clinical trials, observational studies, scientific society recommendations, and public health perspectives, this review aims to offer a comprehensive and clinically applicable overview to support decision-making in maternal and neonatal care.

Accordingly, maternal vaccination against RSV emerges as a strategy to protect infants during the period of greatest immunological vulnerability to infection. The transplacental transfer of neutralizing antibodies seeks to limit the conditions and improve the prognosis by reducing hospitalizations and deaths in the first months of life. In this context, the present work aims to review the literature on maternal vaccination against respiratory syncytial virus, addressing its efficacy, safety, and potential public health impact.

## 2. Methods

This integrative review was conducted to synthesize the current evidence regarding maternal immunization against RSV, focusing on epidemiology, maternal and neonatal clinical implications, prevention strategies, vaccine efficacy and safety, implementation issues, and public health perspectives. A comprehensive literature search was performed in PubMed/MEDLINE, Scopus, Embase, and Google Scholar databases for studies published between January 2010 and June 2026.

The search strategy combined Medical Subject Headings terms and free-text keywords, including: “respiratory syncytial virus”, “RSV”, “maternal immunization”, “maternal vaccination”, “pregnancy”, “pregnant women”, “Abrysvo”, “RSVpreF”, “nirsevimab”, “passive immunization”, “newborn”, “infant”, and “maternal-fetal health”. Boolean operators (“AND”, “OR”) were used to refine the search strategy.

Eligible publications included systematic reviews, meta-analyses, randomized clinical trials, observational studies, narrative reviews, clinical guidelines, recommendations, and position statements issued by national and international scientific societies and regulatory agencies. Articles were included if they addressed maternal RSV immunization, infant passive immunization, vaccine efficacy, safety, implementation strategies, or public health implications. Publications unrelated to maternal or infant RSV prevention, conference abstracts without full-text availability, duplicate records, and studies lacking sufficient methodological or clinical information were excluded. Articles published in English or Portuguese, as well as official documents, guidelines, and position statements issued by national and international scientific societies and regulatory agencies, were considered eligible for inclusion.

Titles and abstracts were independently screened by the authors for relevance. Full texts of potentially eligible studies were subsequently assessed. Additional references were identified through manual review of the bibliographies of selected articles. Given the integrative nature of this review and the heterogeneity of the included sources, a formal risk-of-bias assessment was not performed. Data were extracted and narratively synthesized according to thematic domains, including epidemiology, clinical manifestations, diagnostic approaches, vaccine characteristics, efficacy and safety outcomes, implementation considerations, and preventive strategies.

## 3. Epidemiology of RSV in Pregnant Women and Infants

RSV is one of the main causes of severe lower respiratory tract infections in children worldwide, especially in infants and children under five years of age [8,9]. It is estimated that the pathogen is responsible for about 22% of all acute lower respiratory infections in this age group, resulting in approximately 84,500 to 125,200 deaths annually worldwide [9]. This epidemiology results, above all, from the greater vulnerability of infants, who have still-immature airways and a developing immune system, thus favoring the occurrence of more severe forms of the infection [8].

In addition, RSV constitutes an important cause of hospitalization in infants, with the risk of hospitalization peaking between the first and third month of life and progressively decreasing as the child’s immunological and anatomical maturation occurs [3]. Premature, immunocompromised infants and those with congenital heart or lung disease have a higher risk of presenting more severe forms of the disease. However, it should be noted that most children hospitalized for RSV do not present underlying medical conditions, highlighting the virus’s potential to cause severe disease even in previously healthy children. It is worth noting that RSV infection is associated with important long-term respiratory repercussions, such as reduced pulmonary function and recurrent episodes of wheezing, which may persist into adulthood [9].

Pregnant women also belong to an important risk group, since RSV infections in these women can result in severe complications, such as septic shock, coagulopathy, acute cardiac and renal failure, and severe respiratory complications [2]. In addition, such infection during pregnancy can be associated with high rates of preterm delivery and cesarean section. In this context, it should be noted that the epidemiology of RSV in pregnant women is characterized by an incidence of 26 infected pregnant women per 1000 pregnant women over the course of a year, as well as an attack rate of 10–13% during the respiratory virus season [10,11,12].

## 4. Clinical Manifestations of RSV

The clinical manifestations of RSV infection are quite diverse and generally begin between two and eight days after initial contact. The clinical picture ranges from mild or asymptomatic forms to severe, life-threatening involvement of the lower respiratory tract [11]. In infants, the symptoms are predominantly respiratory, with a characteristic progression from upper to lower airway symptoms. The typical clinical picture includes 2 to 4 days of fever and upper airway symptoms (rhinorrhea, nasal congestion, and sneezing), followed by progression to lower airway symptoms such as tachypnea, nasal flaring, wheezing, and intercostal retractions [13,14]. In addition to these, systemic and behavioral symptoms stand out that demonstrate a decline in the child’s general condition: fever, lethargy, decreased appetite, feeding difficulty, and irritability [15,16]. The most severe cases are concentrated in infants under 6 months of age, who may present more severe inflammation owing to the incomplete development of the airways; in this group, the infection is related to chronic respiratory morbidity, including decreased pulmonary function and recurrent episodes of wheezing that may persist into adulthood [8,10].

In pregnant women, the presentations are generally milder or asymptomatic, but they can cause increased morbidity in pregnant women with underlying comorbidities [17]. The most important maternal complications are septic shock, coagulopathy, acute cardiac failure, acute renal failure, and severe respiratory distress. In addition, such infection is related to an elevated risk of preterm deliveries and higher rates of adverse gestational outcomes [11].

## 5. Diagnosis of RSV

The diagnosis of respiratory syncytial virus infection is based on clinical assessment and laboratory tests, and its importance is justified by the reduction in complementary examinations, of the unnecessary use of antibiotics, and of the length of hospital stay when compared with late diagnosis. The most common clinical manifestations range from mild cold symptoms to severe disease, and its natural history can aid in diagnosis, since it is usually well defined, with nonspecific prodromes for about 2 to 3 days, such as coryza, nasal congestion, low-grade fever, irritability, and loss of appetite. As the disease progresses to the lower airways, the symptoms worsen, and the affected individual may begin to present with a persistent cough, wheezing, tachypnea, intercostal retractions, and nasal flaring. The peak phase occurs between the 3rd and 5th day and usually lasts from 1 to 3 days, with worsening respiratory distress and possible episodes of apnea and hypoxemia in the most severe cases. From this period onward, the disease gradually improves and is usually limited to 14 days. In healthy infants with a typical clinical picture, a purely clinical diagnosis is recommended, since treatment is based on supportive measures and identification of the pathogen does not usually alter management [18]. Imaging examinations can be useful to differentiate bronchiolitis from pneumonia, but they are not necessary and are reserved for cases of diagnostic doubt. The most common findings on bronchiolitis imaging are hyperinflation and focal atelectasis [19].

Owing to the large number of pathogens capable of generating analogous respiratory symptoms and to atypical forms of the disease such as pulmonary involvement or a sepsis-like syndrome, laboratory tests become important for rapid diagnosis in order to organize groups and carry out isolation strategies, but they are not usually recommended routinely [19]. The specific laboratory diagnosis of RSV infection is made by detection of the virus in tissue cultures, of viral antigens by direct and indirect immunofluorescence or by enzyme immunoassays, and by detection of nucleic acid sequences specific to the virus through amplification assays in respiratory secretions. The quality of the sample affects the test result; although samples obtained from nasal washes or nasopharyngeal aspirates yield more sensitive results, the models obtained by nasal swab collection are more comfortable for the patient, require less specialized material, and can be performed in any setting [20].

Viral culture is primarily indicated when laboratory confirmation and viral characterization are required, particularly for epidemiological investigations, strain characterization, and the identification of nosocomial transmission. Although more sensitive than rapid antigen detection tests, it requires specialized laboratory techniques and typically takes 3–6 days to demonstrate the characteristic syncytial cytopathic effect. The advantage of this technique is that it is more sensitive than rapid antigen detection tests and allows for access to viral genetics, favoring studies on the epidemiology and mutation of strains, in addition to identifying nosocomial infections [19].

Tests that detect antigens include direct (DFA) and indirect (EIA) immunofluorescence assays, as well as chromatographic and optical immunoassays. The DFA labels antibodies with a substance called fluorescein, which is able to detect the RSV antigen in the epithelial cells of the respiratory secretion. The advantage of this method over the others is that, in this format, the affected cells can be observed directly by microscopy, which is the main difference from the EIA, in which the RSV antigen is labeled by specific antibodies and detected by a second antibody linked to an enzyme. These tests have a sensitivity of approximately 80% and a specificity of 95 to 98%; however, in adults and older children, the sensitivity is extremely low, since they present lower viral titers. Although they are less sensitive than other methods, the result comes out within minutes, and they are easier to use on an outpatient basis; however, if there is strong clinical suspicion of RSV and the decision depends on the test result, confirmation with RT-PCR is recommended [20].

The main test among the nucleic acid tests (NAT) is the reverse-transcription polymerase chain reaction (RT-PCR), which is considered the gold standard and the method with the highest sensitivity and specificity available, in addition to providing the result within a few hours. The disadvantage of this method is that it requires specialized equipment and knowledge [20]. In PCR-based tests, it is possible to identify the viral load in the affected cells, and although this does not yet have a clear prognostic role, higher viral loads appear to be associated with worse clinical outcomes [19].

The high analytical sensitivity of RT-PCR has substantially improved the diagnosis of RSV infection; however, positive results should always be interpreted within the appropriate clinical and epidemiological context. Viral nucleic acid may be detected in asymptomatic individuals or persist after clinical recovery, particularly in young children. In addition, multiplex PCR platforms have become increasingly important because they enable the simultaneous detection of RSV and other respiratory pathogens, facilitating the identification of viral coinfections and optimizing diagnostic efficiency. These assays support clinical decision-making, infection control measures, and antimicrobial stewardship, particularly in hospitalized patients and during periods of increased respiratory virus circulation [19].

Thus, the diagnosis of RSV is based on the correlation between clinical and laboratory findings. In most typical cases, the patient’s signs and symptoms are sufficient to guide medical management; however, in specific situations that require closer surveillance, such as immunocompromised patients and hospital outbreaks, laboratory tests can be valuable allies (Table 1).

## 6. Analyses by Regulatory Agencies Worldwide

In 2023, the FDA (Food and Drug Administration) approved Pfizer’s Abrysvo^®^, a direct-immunization vaccine administered as a single 0.5 mL intramuscular dose, for pregnant women with a gestational age of 32 to 36 weeks [8,9,21,22], to transfer antibodies to the fetus via the placenta, as a means of preventing neonatal complications of respiratory syncytial virus, demonstrating 80% efficacy in newborns up to 90 days of age [21]. However, the agency warns about the decline in vaccine efficacy in cases of prematurity, since the time available for antibody transfer from the mother’s vaccine to the infant is reduced. The FDA also includes older adults over 60 years of age in vaccination [11], considering that this population is also at risk owing to immunosenescence and comorbidities.

In Brazil, the National Health Surveillance Agency (Anvisa) approved, in 2024, the Abrysvo^®^ vaccine for maternal immunization against RSV. The indication covers pregnant women between 24 and 36 weeks of gestation, with the objective of conferring passive protection to newborns against lower respiratory tract infections associated with RSV during the first months of life. Subsequently, in 2025, the Ministry of Health incorporated the vaccine into national prevention strategies, expanding access to maternal immunization in the country and aligning Brazil with international recommendations for the reduction in RSV-related infant morbidity and mortality [23,24] (Table 2).

## 7. Mechanism of Action of the Abrysvo Vaccine

RSVpreF (respiratory syncytial virus pre-fusion F protein), commercially known as Abrysvo, is a recombinant and bivalent vaccine produced by Pfizer [25]. It is approved for administration in pregnant women with a gestational age between 32 and 36 completed weeks and ensures passive immunization of the fetus against RSV at birth [8]. This vaccine contains fusion F glycoproteins of both antigenic subgroups of the virus, RSV A and RSV B, stabilized in their prefusion (pre-F) form [8,22]. The RSV F protein is intrinsically unstable and tends to alter its structure rapidly; in order to keep the pre-F form stable, Pfizer uses an optimized construct that incorporates stabilizing mutations into the structure of the glycoprotein, which increase conformational stability and prevent premature transition to the postfusion form [26]. This structural modification aims to preserve the antigenic sites (0, I, II, III, IV, V) of the pre-F glycoprotein, mainly epitopes zero and five, which are lost with the transition to the postfusion form [27]. These are exclusive to the pre-F form and have high neutralizing activity, in addition to promoting the activation and proliferation of memory B cells, which leads to a superior immune response and results in a more substantial transfer of maternal antibodies to the fetus [27,28].

Once administered, the stabilized pre-F antigens reach the maternal circulation and trigger an immune response and the production of maternal immunoglobulin G (IgG). The transplacental transfer of maternal IgG is mediated primarily by the neonatal Fc receptor (FcRn), which is highly expressed on syncytiotrophoblast cells of the chorionic villi. FcRn binds maternal IgG within acidic endosomal compartments and actively transports these antibodies across the placental barrier into the fetal circulation, where they are released under physiological pH conditions. This process intensifies progressively throughout gestation, particularly during the third trimester, explaining why maternal vaccination is recommended between 32 and 36 weeks of gestation. Several factors influence the efficiency of placental antibody transfer, including gestational age at vaccination, the interval between vaccination and delivery, prematurity, placental dysfunction, maternal infections, hypertensive disorders, and other conditions associated with impaired placental function. In addition, antibody-specific characteristics, such as IgG subclass distribution and Fc glycosylation profiles, may also affect FcRn binding affinity and transfer efficiency. These factors may contribute to interindividual variability in neonatal antibody concentrations and, consequently, in the degree of passive protection conferred to the infant [8,10,29,30,31,32].

## 8. Schedule and Dosage

Maternal vaccination against RSV constitutes an effective strategy for the prevention of RSV-associated lower respiratory tract disease in infants in the first months of life. Currently, the vaccine approved for use during pregnancy is the bivalent prefusion F protein vaccine (RSVpreF), marketed as Abrysvo^®^ [25]. The formulation was developed from the stabilization of the F protein in its prefusion conformation, which enables greater induction of neutralizing antibodies compared with formulations based on the postfusion protein [26,27].

The RSVpreF vaccine (Abrysvo^®^) is recommended for pregnant women between 32 weeks and 36 weeks and 6 days of gestation, administered as a single 0.5 mL dose by the intramuscular route [8,22]. The definition of this gestational window took into account the results of clinical trials and the safety data evaluated by the regulatory agencies [22]. In addition, vaccination during this period allows for adequate transplacental transfer of maternal antibodies to the fetus, promoting protection against severe respiratory infections caused by RSV during the first months of life [10,29].

## 9. Efficacy, Effectiveness, and Safety of the Vaccine

The Abrysvo vaccine, when administered to pregnant women, demonstrated impactful efficacy in preventing severe RSV lower respiratory tract infections (LRTI) [28]. In the phase 3 clinical trial, the MATISSE Study, about 3682 pregnant women were vaccinated, and 3676 formed the placebo group. The group that received Abrysvo had the occurrence of 6 severe infection events (0.16%) over a period of 90 days after birth, while in the group that received placebo there were 33 events (0.99%), thus demonstrating an efficacy of 81.8% (99.5% CI: 40.6–96.3) [25,28]. In addition, over the period of 180 days following birth, 19 cases of LRTI were recorded in the vaccinated group (0.51%), while in the other group there were 62 events (1.68%). Thus, the Abrysvo^®^ vaccine provides protection with an efficacy of 69.4% (97.58% CI: 44.3–84.1) up to 180 days after birth [11,25]. Furthermore, maternal vaccination demonstrated a large reduction in the number of hospitalizations for laboratory-confirmed RSV. According to the Cochrane review, there was a reduction of about 11 fewer hospitalizations for every 1000 newborns of vaccinated mothers, with prevention of 50% of admissions (RR 0.50) [27]. Complementarily, molecular studies indicate the molecular superiority of vaccines based on the F protein in its prefusion state, which preserves critical epitopes, by inducing a greater immune response with significant neutralizing potential [26,27].

The adverse effects related to the vaccine are frequent, although the majority are of mild to moderate intensity and transient in nature [25]. The predominant symptoms include hyperemia, edema, and pain at the injection site, headache, fatigue, and myalgia [25]. Compared with placebo, vaccination presented almost no real risk of congenital anomalies (RR 0.96; 95% CI: 0.88–1.04; high level of certainty). Likewise, there was no impact on intrauterine growth restriction (RR 1.32; 95% CI: 0.75–2.33; moderate level of certainty) or on stillbirth (RR 0.81; 95% CI: 0.38–1.72; low level of certainty) [29].

A numerical imbalance in preterm birth rates was observed in the MATISSE trial, with preterm delivery occurring in 5.7% of vaccinated participants compared with 4.7% in the placebo group [29]. However, this difference did not reach statistical significance, and no causal relationship between maternal RSV vaccination and preterm birth has been established. Although this imbalance was observed predominantly in middle- to low-income countries, regulatory agencies adopted a precautionary approach and recommended vaccine administration between 32 and 36 weeks of gestation [28]. Post-marketing surveillance data from the Vaccine Safety Datalink (VSD) subsequently demonstrated a preterm birth incidence of 4.1% among vaccinated pregnant women, which falls within the expected range for the general population (3.1–6.1%) [28,29,30,31,32].

Similarly, slight numerical differences in hypertensive disorders, including preeclampsia, were observed between study groups. In the phase 3 trial, preeclampsia occurred in 1.8% of vaccinated participants compared with 1.4% in the placebo group [28]. However, these differences were not statistically significant, and no causal relationship between maternal RSV vaccination and hypertensive disorders has been established. Post-marketing surveillance data from the CDC also identified slightly higher rates of hypertensive disorders among vaccinated pregnant women; however, subsequent analyses suggested that these findings were likely influenced by confounding factors rather than representing a vaccine-related safety signal [28,29,30,31,32].

## 10. Adverse Effects

Safety and efficacy are aspects evaluated in the development of vaccines intended for gestational use. In addition to the protection conferred, it is necessary to ensure that the substance does not generate significant maternal, fetal, or neonatal adverse events. Pfizer’s clinical trial of the maternal RSVpreF vaccine studied 7358 pregnant women between 24 and 36 weeks of gestation, of whom 3682 received the vaccine and the others the placebo. The study concluded that the vaccine was effective in preventing severe RSV-associated infections that required medical care, with no safety problems identified in relation to the vaccination [33].

One study raised the hypothesis of an association between vaccination and the occurrence of preterm deliveries, possibly related to the inflammatory response triggered by the vaccination process [34]. To further evaluate maternal and infant safety, the MATISSE study (Maternal Immunization Study for Safety and Efficacy), a randomized placebo-controlled trial involving more than 7000 pregnant women, demonstrated high efficacy in preventing severe RSV-associated lower respiratory tract disease during early infancy and showed no major safety signals in mothers or infants up to 24 months of follow-up [35]. Most newborns were delivered at term and had adequate birth weight, and no significant differences were observed between groups regarding low birth weight, neonatal hospitalization, or other important neonatal outcomes. However, when preterm delivery was specifically analyzed, it occurred in 5.7% of vaccinated women compared with 4.7% in the placebo group, corresponding to an approximately 20% increase in relative risk. Importantly, this difference was not statistically significant, as the 95% confidence interval (95% CI: 0.98–1.46) included the null value, precluding the establishment of a causal association between maternal RSV vaccination and preterm birth. Nevertheless, as a precautionary measure, regulatory agencies recommended vaccine administration between 32 and 36 weeks of gestation to further minimize any potential risk [30].

The most frequent local maternal adverse reactions are redness, swelling, and pain at the application site. With regard to systemic effects, some pregnant women presented headache, joint pain, fever, gastrointestinal disturbances, fatigue, and, less frequently, cardiac disturbances, especially in the first month after vaccination. However, the only symptom observed with significantly greater frequency among vaccinated women compared with the placebo group was muscle pain. In infants, the participants whose mothers received the vaccine and those whose mothers received placebo presented similar results with regard to the occurrence of adverse events, with no clinically relevant differences between the groups [34].

In general, the available studies demonstrate that maternal vaccination against RSV presents a favorable safety profile, with predominantly mild and transient adverse events. Although the possible association with preterm delivery remains under monitoring, the current evidence indicates that the benefits of immunization in preventing severe forms of RSV infection outweigh the potential risks observed to date [34].

## 11. Economic Analysis

RSV is responsible for a high demand for healthcare services, such as outpatient visits, emergency services, and admissions to pediatric intensive care units [22,25]. About 2% to 3% of infants require hospitalization for RSV, and there are 58,000 to 80,000 annual RSV-associated hospitalizations in children under 5 years of age [36,37]. Although most cases present a mild clinical course, a significant proportion of patients progress with involvement of the lower respiratory tract, resulting in bronchiolitis, pneumonia, recurrent wheezing, and chronic respiratory diseases such as asthma. For this reason, preventive strategies have been evaluated not only from a clinical perspective but also from an economic one. The reduction in severe cases and hospitalizations observed in clinical studies suggests a potential impact on decreasing the costs related to disease management, especially those associated with medium- and high-complexity hospital care.

Among the included studies, the analysis carried out by the Advisory Committee on Immunization Practices (ACIP) provided the most detailed economic data. The results demonstrated that the cost-effectiveness ratio of maternal vaccination is influenced by the way the strategy is implemented. When year-round vaccination was considered, a less favorable incremental cost-effectiveness ratio was observed compared with seasonal vaccination. Administration concentrated in the periods of greatest viral circulation presented better economic performance, indicating greater efficiency in the use of available resources, with USD 167,280 per QALY gained from September to January; thus, the seasonal strategy was considered more efficient [36].

Another relevant aspect concerns the comparison between maternal vaccination and passive immunization by monoclonal antibodies. Although nirsevimab demonstrates significant efficacy in preventing severe disease caused by RSV, its use requires resources allocated to the acquisition, storage, transport, and administration of the immunobiological [21,38]. In contrast, vaccination during pregnancy uses the already-established structure of prenatal care services, potentially requiring less logistical investment.

In addition, the economic viability of the strategy depends on local epidemiological characteristics, including viral seasonality, disease incidence, vaccination coverage achieved, and availability of financial resources. The European Board and College of Obstetricians and Gynaecologists (EBCOG) position statement emphasizes that decisions related to the incorporation of the vaccine into national programs should simultaneously consider evidence of effectiveness, implementation costs, and the operational capacity of health systems [9]. Similarly, the Brazilian College of Obstetrics and Gynecology (FEBRASGO) document emphasizes that the high burden of disease associated with RSV justifies the evaluation of preventive measures capable of reducing the use of hospital resources and improving care efficiency [39].

## 12. Seasonal Administration of the RSVpreF Vaccine

RSV exhibits marked seasonal variation, although its epidemiological patterns differ substantially across geographic regions. In temperate climates, RSV circulation typically peaks during the autumn and winter months, whereas in tropical and subtropical regions viral activity may occur throughout much of the year or coincide with rainy seasons rather than colder temperatures. Although RSV transmission is considerably lower outside peak seasons, sporadic infections continue to occur year-round. Environmental factors, including temperature, humidity, rainfall, population density, and air pollution, have been associated with variations in RSV transmission, disease severity, and hospitalization rates. In addition, behavioral factors, including school attendance, daycare exposure, and school holiday periods, may influence transmission dynamics by altering contact patterns among young children, who serve as the primary reservoir for community spread. The marked disruption of RSV seasonality observed during the COVID-19 pandemic further highlighted the importance of population mobility and social mixing in determining the timing and intensity of RSV epidemics. These regional and behavioral differences underscore the importance of adapting maternal vaccination strategies to local epidemiological patterns rather than relying on a single universal seasonal schedule.

The RSVpreF vaccine was developed to be administered during pregnancy, allowing for the transplacental transfer of maternal antibodies to the fetus. Thus, the newborn presents protective levels of antibodies in the first months of life, the period in which there is greatest vulnerability to severe forms of RSV infection [21,22]. Given that the protection conferred by vaccination has a limited duration, the definition of the ideal time of administration is an important factor for maximizing the benefits of vaccination.

The guidelines of the Advisory Committee on Immunization Practices (ACIP) report that maternal vaccination with RSVpreF should be carried out between 32 and 36 weeks of gestation during the RSV circulation season. This strategy ensures that the antibodies transferred to the fetus are at ideal concentrations at birth and during the first months of life, coinciding with the period of greatest viral activity in the community [22], since seasonal administration makes it possible to direct the intervention to pregnant women whose newborns will be most exposed to the virus after birth.

According to the EBCOG, the implementation of vaccination should consider the epidemiological particularities of each region, since the seasonality of RSV may vary between distinct countries and geographic areas [9]. The definition of the best periods for vaccination requires continuous monitoring of local viral circulation, allowing for adaptations to the specific characteristics of each health system. In temperate-climate regions, viral circulation occurs predominantly during the colder months of the year, usually occurring between October and March in most of the United States; to align and maximize the protection of the newborn, the RSVpreF vaccine is administered between September and January [22].

In Brazil, being a predominantly tropical country, the dynamics of RSV differ from those observed in temperate-climate countries. Owing to the climatic variations among the regions, immunization strategies should not be uniform throughout the country, and regional adaptation of immunization and prophylaxis programs becomes necessary. Based on the epidemiological patterns identified, in the North, Central-West, and Southeast regions, immunoprophylaxis should be started in January, considering that the peak of viral circulation tends to occur in the subsequent months. In the Northeast region, the recommended start would be in February, following the local seasonality pattern. In the South region, where RSV circulation occurs later, immunization should begin in March, preceding the epidemic peak generally observed during the winter [40]. Although these recommendations were originally developed for the use of immunoprophylaxis in risk groups, the epidemiological principles remain the same for maternal vaccination against RSV [9,40].

## 13. Simultaneous Administration with Other Vaccines

The concomitant administration of the RSVpreF vaccine with other vaccines recommended during pregnancy is considered safe and may contribute to increasing maternal vaccination coverage [22,28]. According to the recommendations, the RSV vaccine can be administered simultaneously with the vaccines recommended during pregnancy, including the vaccines against influenza, COVID-19, and acellular diphtheria, tetanus, and pertussis (Tdap), with no need for minimum intervals between applications [22,28].

The coadministration of these vaccines presents important advantages, such as the reduction in the number of visits needed for immunization and the increase in adherence to the prenatal vaccination schedule [28]. To date, there is no evidence of clinically significant interference in the immune response or in the safety profile when the RSV vaccine is administered concomitantly with the other vaccines recommended during pregnancy [22,28]. Thus, simultaneous administration constitutes a practical and safe strategy for expanding maternal and neonatal protection against important vaccine-preventable infectious agents [22].

## 14. Additional Doses of the Vaccine in Subsequent Pregnancies

Currently, there is insufficient scientific evidence to determine the safety of administering additional doses of Abrysvo^®^ in subsequent pregnancies, which is why revaccination is not recommended by bodies such as the American College of Obstetricians and Gynecologists (ACOG) and the EBCOG [9,28]. Accordingly, healthcare professionals should rigorously review the patient’s records and vaccination history before prescribing Abrysvo^®^ [28]. In addition, the CDC reinforces that there are also insufficient data to indicate to us that the immune protection conferred to the fetus of the pregnancy in which the vaccine was administered will be maintained in subsequent pregnancies, since passive maternal immunity is temporary and antibody titers tend to decline after delivery [29]. Therefore, to ensure neonatal safety in new pregnancies, the protection of newborns should be carried out through direct immunization with monoclonal antibodies, such as nirsevimab [28,29].

## 15. Precautions and Contraindications

The RSVpreF vaccine presents a favorable safety profile for use during pregnancy [10,29]. However, some precautions should be observed. The main one concerns the exclusive administration between 32 weeks and 36 weeks and 6 days of gestation, since clinical studies identified a possible numerical increase in preterm deliveries among vaccinated pregnant women when compared with the control groups. Although a definitive causal relationship has not been established, the regulatory authorities adopted this gestational window as a safety measure [8,22].

The main adverse events in the study were preterm deliveries, low birth weight, and developmental delay. The most frequent conditions observed in the pregnant mother were preeclampsia, characterized by elevated blood pressure and the inherent risks of fetal distress syndrome secondary to hypoxia in the fetus. Another adverse effect in the first months of life was neonatal jaundice [8]. Vaccination should be postponed in pregnant women who present with moderate or severe acute illness until resolution of the clinical condition [28].

The main contraindication of the vaccine consists of a previous occurrence of a severe hypersensitivity reaction, including anaphylaxis, to any component of the vaccine formulation [22,28]. To date, no other specific contraindications have been described for pregnant women beyond the recommendations related to the appropriate gestational age for administration [8]. Considering the available data, the benefits of maternal vaccination in preventing severe RSV disease in infants outweigh the potential risks identified in clinical studies [10,29].

## 16. Studies on the Development and Efficacy of the RSV Vaccine in Infants and Children

The development of RSV vaccines for use in infants and children remains a major challenge in pediatric vaccination. Vaccine strategies in adults and pregnant women already have results approved for clinical use, but vaccines intended for infants and children have been limited by safety concerns related to the initial immune response to the virus [21,41].

The formulation of pediatric RSV vaccines was affected by the results obtained in the 1960s with the formalin-inactivated RSV (FI-RSV) vaccine. After subsequent exposure to the circulating virus, previously vaccinated children presented more severe respiratory conditions than non-immunized children, a phenomenon later termed RSV-associated enhanced respiratory disease [21,41,42].

Research showed that formalin inactivation alters the structure of important viral glycoproteins, reducing the capacity to generate high-affinity neutralizing antibodies. As a result, an insufficient immune response was observed, characterized by the formation of immune complexes and by an inflammatory pattern, mainly mediated by type 2 helper T lymphocytes (Th2). These factors are associated with the development of RSV-associated enhanced respiratory disease [21,41,42]. Similar observations were reported in experimental studies with formalin-inactivated parainfluenza virus vaccines (FI-PIV), corroborating the idea that the structural changes resulting from the inactivation process can affect the quality of the induced immune response [41].

After the events related to the FI-RSV vaccine, the development of new immunizing agents began to prioritize strategies capable of conferring protection without triggering immunopathological responses. At present, studies are concentrated on four main technological strategies: live-attenuated virus vaccines, vaccines based on viral particles or virus-like particles (VLPs), protein subunit vaccines, and vaccines that use recombinant viral vectors [21,42]. Each of these platforms presents particularities with regard to immunogenicity, safety profile, and suitability for the various pediatric age groups, which highlights the complexity of developing RSV vaccines in childhood.

Several pediatric RSV vaccine candidates are currently undergoing advanced clinical development. Among the most promising approaches are live-attenuated vaccines, which are particularly attractive for young infants because they can induce mucosal, humoral, and cellular immunity while minimizing the risk of vaccine-enhanced disease. In addition, vector-based vaccines, such as ChAd155-RSV, have demonstrated favorable safety and immunogenicity profiles in clinical trials involving seropositive children. Other candidates under investigation include protein subunit vaccines and VLP-based formulations. Although none of these vaccines has yet been approved for routine pediatric use, ongoing phase 2 and phase 3 studies are expected to further clarify their efficacy, safety, and potential role within future RSV prevention strategies [43,44].

The structural characterization of the RSV fusion protein (F) in its prefusion (pre-F) conformation represented significant progress in the development of new vaccines. This structure presents highly neutralizing epitopes, which enable the induction of more intense humoral responses than those produced by the postfusion conformation. This understanding allowed for the development of safer and more immunogenic vaccines, serving as the foundation for several strategies currently under investigation [42,45].

In response to the limitations identified in inactivated vaccines, current vaccine development programs prioritize strategies considered safer for children. According to research on vaccines under development, the main approaches studied for children who have never been exposed to RSV are live-attenuated virus vaccines, intranasal chimeric vaccines, and vaccines based on recombinant viral vectors [21,42]. These strategies aim to replicate elements of natural infection in a controlled manner, activating humoral and cellular immune responses, without the dangers associated with the formulations previously employed [41].

Among the candidates submitted to clinical evaluation is the vaccine that uses the adenoviral vector ChAd155-RSV. In the research carried out by Díez-Domingo et al. [45], which included healthy seropositive children aged between 12 and 23 months, the vaccine demonstrated a positive safety profile and significantly increased the levels of neutralizing antibodies and the indicators of cellular immunity. These findings highlight the potential of vector technologies as a promising strategy to increase protection against RSV in childhood.

In addition to the creation of active vaccines, important progress was made with passive immunization strategies aimed at infants. For more than 20 years, palivizumab was the main preventive strategy against RSV, but its use was limited to high-risk pediatric groups, owing to the need for continuous administrations during the viral season [21]. The search for more complete options led to the development of long-acting monoclonal antibodies, resulting in the approval of nirsevimab in 2023. This antibody has a long half-life and high neutralizing capacity against several RSV strains, which enables prolonged protection with a single application and expands the prevention options to a larger number of infants [21]. These advances demonstrate that, although the development of active pediatric vaccines still faces challenges related to safety and the immunological maturation of early childhood, passive immunization strategies already constitute an important tool for reducing the burden of disease caused by RSV [21,42].

Although passive immunization remains the main prevention strategy in the first months of life, advances in the understanding of viral structure and the immune response have driven the development of increasingly safe and effective pediatric vaccines. Thus, the current view is that future active immunization strategies may complement existing measures and offer more lasting protection during childhood [21,41,42,45] (Table 3).

## 17. Studies on Maternal Vaccination Against RSV During Pregnancy

Maternal vaccination against RSV represents a relevant preventive strategy for protecting infants in the first months of life. Based on vaccines using the F protein in the prefusion conformation, this approach induces an immune response in the pregnant woman, increasing the production of specific antibodies and promoting their placental transfer to the fetus [8,41]. Thus, the newborn receives passive protection from birth, the period of greatest vulnerability to severe respiratory infections.

Determining the ideal moment for the administration of maternal vaccination is fundamental for its efficacy. Considering that the placental transfer of IgG antibodies intensifies in the third trimester of pregnancy, the application of the vaccine is recommended between the 32nd and the 36th week. This interval favors the adequate production of maternal antibodies and their efficient transfer to the fetus before delivery, contributing to the protection of the newborn in the first months of life [11,28].

Several clinical trials, systematic reviews, and observational studies support the efficacy of maternal vaccination. The Cochrane systematic review conducted by Phijffer et al. [29] demonstrated that vaccination during pregnancy significantly increases the levels of specific antibodies transferred to the newborn and is associated with a reduction in adverse outcomes related to RSV infection in the first months of life. Subsequent clinical studies and opinions from scientific societies also confirmed the favorable safety profile of the vaccine during pregnancy, which favored its inclusion in the current maternal immunization recommendations [8,11,28,29].

In addition to the results from controlled studies, real-world evidence has expanded the understanding of the population-level impact of maternal vaccination. The multicenter BERNI study [46], carried out in Argentina, demonstrated a significant reduction in hospitalizations for RSV-associated respiratory infections in infants whose mothers received the vaccine during pregnancy. Scruzzi et al. [47] reported similar findings, reinforcing the efficacy of the strategy in routine clinical practice settings. The consolidation of this evidence supported the inclusion of maternal vaccination against RSV in the recommendations of scientific societies and regulatory bodies. In Brazil, FEBRASGO began to recommend vaccination during pregnancy [11], aligning itself with the guidelines of the ACOG [28] and the EBCOG [9].

The current evidence indicates that maternal vaccination is one of the most effective strategies for reducing the incidence of RSV-related diseases in infants. This approach complements other preventive measures and contributes to the reduction in hospitalizations and respiratory complications in the first months of life [9,10,11,28,29,41,46,47]. Despite the demonstrated efficacy and safety of maternal RSV immunization, successful implementation depends on several factors beyond vaccine availability. Vaccine acceptance during pregnancy remains a major determinant of program effectiveness. Vaccine hesitancy, frequently influenced by concerns regarding fetal safety, misinformation, lack of awareness, and insufficient counseling by healthcare professionals, may significantly reduce vaccine uptake. Evidence from maternal immunization programs consistently demonstrates that healthcare provider recommendation is one of the strongest predictors of vaccine acceptance during pregnancy.

Socioeconomic disparities also play an important role in access to maternal immunization. Women from socially vulnerable populations, particularly those living in low- and middle-income countries (LMICs), rural areas, or underserved communities, often experience reduced access to prenatal care and vaccination services. Since the burden of severe RSV disease is frequently highest in LMICs, these inequities may further amplify existing health disparities. In addition, financial considerations, including vaccine cost, reimbursement policies, and the availability of public funding, may substantially influence implementation strategies across healthcare systems.

From a health policy perspective, successful integration of maternal RSV vaccination into routine prenatal care requires coordinated public health strategies, including clear national recommendations, healthcare professional education, surveillance systems, and incorporation of vaccination into standard antenatal care pathways. The coadministration of RSV vaccine with other recommended maternal vaccines, such as influenza, Tdap, and COVID-19 vaccines, may further facilitate implementation and improve vaccination coverage. Addressing these operational and social challenges will be essential to maximize the population-level benefits of maternal RSV immunization.

## 18. Maternal Immunization Versus Infant Immunization Against RSV

Currently, the prevention of RSV infection in infants is based on two main strategies: vaccination of the mother during pregnancy and administration of monoclonal antibodies to the newborn. Both seek to reduce severe cases in the first months of life, but they differ with regard to the biological mechanisms, the timing of application, and the groups served [21]. When the mother is vaccinated, she produces specific antibodies that are transmitted to the infant through the placenta. Thus, the newborn is already born protected, taking advantage of the natural transfer of IgG immunoglobulins during pregnancy [29,48]. In contrast, immunization of the infant with monoclonal antibodies directly provides neutralizing antibodies produced by biotechnology [21].

One of the main differences between the strategies, from a clinical point of view, concerns the dependence on gestational age. The effectiveness of maternal vaccination is linked to the adequate transfer of antibodies through the placenta, which occurs mainly in the third trimester of pregnancy. Thus, preterm deliveries can reduce the period available for the passive transfer of immunity to the fetus [11,28,29]. On the other hand, it is possible to administer monoclonal antibodies directly to the newborn after delivery, which represents an especially important alternative for premature infants or for infants whose mothers were not vaccinated during pregnancy [21,48].

Effectiveness studies conducted in clinical practice settings demonstrate substantial benefits for both strategies. Evidence from the BERNI study [46] and from observational analyses carried out by Scruzzi et al. [47] indicates a significant reduction in hospitalizations for RSV-associated respiratory infections after the introduction of maternal vaccination. In addition, monoclonal antibodies continue to play a fundamental role in protecting groups with greater clinical vulnerability and in contexts in which maternal immunization was not carried out [21,42]. For this reason, the studies show that these strategies should be seen as complementary, and not as competing. Maternal RSV vaccination should be considered the preferred preventive strategy for most pregnant women when administered within the recommended gestational window, as it provides passive protection from birth through transplacental antibody transfer. However, nirsevimab remains particularly relevant for specific clinical situations, including infants born prematurely, infants delivered less than 14 days after maternal vaccination, infants born to unvaccinated mothers, or infants whose mothers have contraindications to vaccination. In addition, infants at increased risk for severe RSV disease may particularly benefit from passive immunization strategies. Therefore, maternal vaccination and nirsevimab should be viewed as complementary rather than competing interventions. Their combined use, when clinically indicated and supported by national recommendations, may maximize protection against severe RSV disease during early infancy. Using the two strategies together is one of the best ways to reduce the impact of disease caused by RSV in the first months of life [9,10,21,41,42,46,47,48].

### 18.1. Impact of Maternal Vaccination on Neonates

Maternal vaccination against RSV is an important strategy for the prevention of severe respiratory infections in neonates, especially in the first six months of life, the period in which the infant’s immune system is still immature [8]. The main mechanism responsible for this protection is the transplacental transfer of neutralizing maternal antibodies (IgG), produced after immunization of the pregnant woman during the third trimester of pregnancy [8]. These antibodies cross the placenta and enter the fetal circulation, conferring passive immunity to the newborn [8,10]. Studies show that this transfer is more effective when vaccination occurs at least 14 days before delivery, with a significantly higher concentration of antibodies observed in neonates when the interval between vaccination and birth exceeds 5 weeks, since this moment is chosen to maximize the placental transfer of IgG via the FcRn receptor, which increases in the third trimester, and to provide high titers of specific antibodies to the newborn during the period of greatest vulnerability [22,49].

The benefits of administering the vaccine to pregnant women were evidenced in the phase 3 MATISSE clinical trial, which evaluated the efficacy of the RSVpreF vaccine in pregnant women. The results showed important efficacy in preventing severe lower respiratory tract diseases associated with RSV in the first 90 days of the infant’s life, maintaining protection of approximately 70% up to 180 days after birth. Accordingly, a significant reduction was observed in hospitalizations, the need for oxygen therapy, and admissions to neonatal intensive care units related to respiratory complications caused by RSV [8,50].

**Figure 1:**
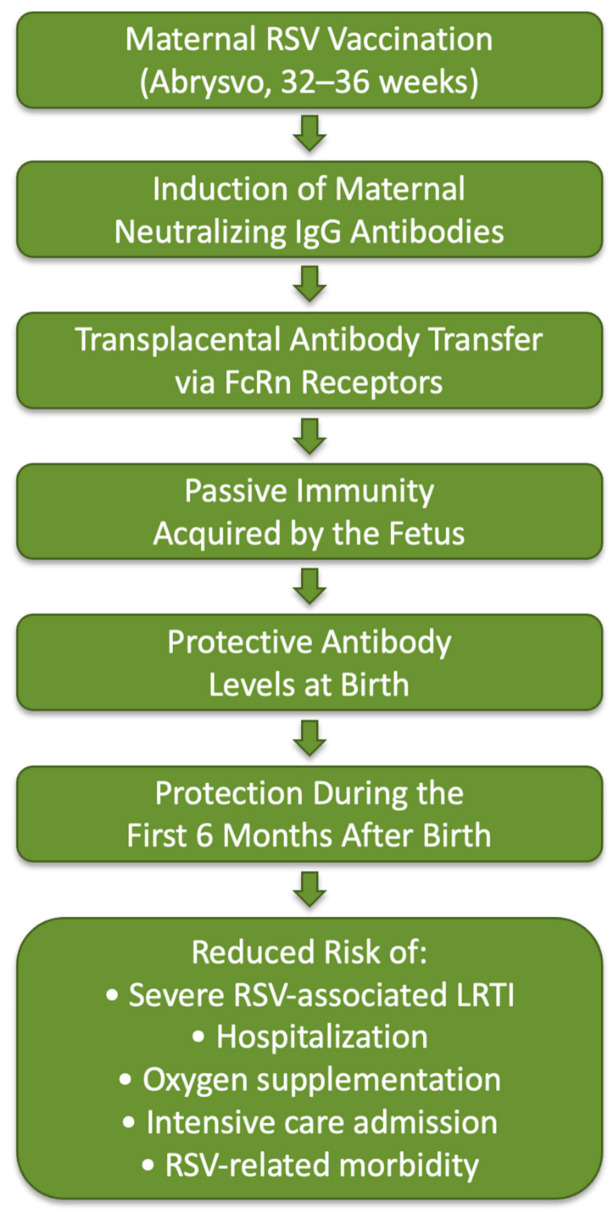
Mechanism of protection conferred by maternal RSV vaccination. Maternal immunization with the respiratory syncytial virus prefusion F protein vaccine (RSVpreF vaccine; Abrysvo^®^) induces the production of maternal neutralizing immunoglobulin G (IgG) antibodies, which are actively transported across the placenta through neonatal Fc receptors (FcRn). This process provides passive immunity to the fetus, resulting in protective antibody levels at birth and reducing the risk of severe respiratory syncytial virus-associated lower respiratory tract infection (LRTI), hospitalization, oxygen supplementation, intensive care admission, and RSV-related morbidity during the first six months of life.

Despite the expressive benefits, some studies opened discussions regarding the safety of vaccination during pregnancy. In the clinical trials, a slight numerical increase was identified in the incidence of preterm deliveries among vaccinated pregnant women compared with the placebo group, although without consistent statistical significance across all analyses. Owing to this possible risk, regulatory bodies such as the FDA recommended administering the vaccine preferentially between 32 and 36 weeks of gestation, aiming to minimize the occurrence of extreme prematurity. Slightly higher incidences of neonatal jaundice and low birth weight were also described, events frequently associated with cases of preterm birth. Even so, the final analyses of the MATISSE study demonstrated a favorable safety profile for both pregnant women and neonates, with no identification of important new risk signals [8,22] (Figure 1).

### 18.2. Passive Immunization in Infants and Children

Passive immunization is one of the main strategies for preventing infection in infants and young children. Unlike active vaccination, which stimulates the production of antibodies by the organism itself, passive immunization provides specific antibodies against the virus, offering immediate protection against infection, without depending on the immune response of the infant or child. It is of extreme value in the first months of life, the moment in which infants present greater susceptibility to severe forms of the disease and still have an immature immune system [21,49].

Palivizumab was the main alternative for preventing the disease in high-risk children for a long time. However, there are limitations related to the short duration of protection, requiring monthly applications throughout the viral circulation season. The development of nirsevimab represented an advance, since the antibody was designed to have a long half-life, ensuring protection throughout the rise in RSV after a single administration [49,51].

Accordingly, the administration of a single dose facilitates adherence to prevention programs, reduces the need for multiple visits to healthcare services, and expands the potential for population coverage during the viral season. These factors have contributed to the incorporation of this strategy in several countries as a protective measure during the first season of exposure to RSV [51,52].

Both maternal vaccination and passive immunization with monoclonal antibodies present high efficacy in preventing severe disease caused by RSV. However, there are some relevant differences between the strategies. Maternal vaccination offers the advantage of conferring protection from birth through transplacental antibody transfer; however, the process requires approximately 14 days after administration of the vaccine; that is, infants born less than two weeks after maternal vaccination may not receive sufficient levels of protective antibodies, and the administration of nirsevimab is recommended in these cases. In addition, preterm newborns under 34 weeks of gestational age are also candidates for passive immunization, since they may not have benefited from the placental transfer of maternal antibodies [21,53,54].

Although nirsevimab has demonstrated excellent effectiveness in preventing RSV-associated hospitalization and severe lower respiratory tract infection in real-world studies, recent surveillance has identified rare breakthrough infections associated with amino acid substitutions in the RSV fusion (F) protein that reduce susceptibility to monoclonal antibody neutralization. Although these variants remain uncommon and current evidence indicates that nirsevimab continues to provide substantial clinical protection, ongoing genomic surveillance is essential to monitor the emergence of resistant strains and to evaluate their potential impact on the long-term effectiveness of passive immunization strategies. These findings highlight the importance of maintaining complementary prevention approaches, including maternal vaccination, rather than relying on a single intervention [55,56].

**Figure 2:**
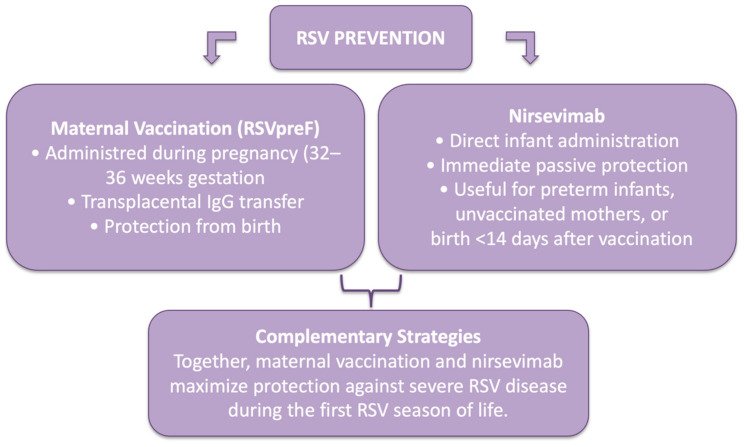
Prevention strategies against RSV in early infancy. Maternal RSV vaccination and infant immunization with nirsevimab should be viewed as complementary rather than competing strategies. Maternal vaccination provides passive protection from birth through transplacental antibody transfer, whereas nirsevimab offers direct protection to infants who may not have received sufficient maternal antibodies, such as preterm infants, infants born shortly after maternal vaccination, or infants of unvaccinated mothers. Together, these approaches maximize protection against severe RSV disease during the first months of life. Abbreviations: RSV, respiratory syncytial virus; RSVpreF, respiratory syncytial virus prefusion F protein vaccine; IgG, immunoglobulin G.

The preventive strategy does not depend only on the clinical and gestational angle, but also on perspectives related to health systems. Among them are access to healthcare services, the availability of immunobiologicals, and the timing of birth in relation to viral circulation [21,22,51] (Figure 2).

The main practical recommendations for healthcare professionals regarding maternal RSV immunization are summarized in Table 4.

## 19. Factors Influencing Immunization Strategies

The strategy for immunization against neonatal respiratory syncytial virus varies according to the particular clinical history of pregnant patients or mothers of young infants. The preferable scenario would be vaccination of the pregnant woman in the third trimester, thus ensuring fetal immunization still in utero [8,9,21,22]. However, low adherence to vaccination and prenatal care, triggered by factors such as misinformation, lack of coverage and campaigns, and socioeconomic and cultural situations, prevents certain groups from accessing the vaccine [21]. In addition, the cases in which the pregnant woman adhered to active immunization but had a preterm delivery (between the 29th and 32nd week) stand out, causing its efficacy to fall [11,21]. For both, a postnatal strategy is chosen.

In newborn infants, passive immunization is used, applied about one month before the respiratory syncytial virus season, which varies according to the geographic location. For example, in the Southeast of Brazil it occurs between the months of April and May, and in the Northeast during the rainy seasons [11]. The child will be immunized only in the first season of the virus, before completing one year of age. Nirsevimab is the most recommended antibody for this situation, being a single application. Palivizumab, in turn, is chosen for premature children, those with congenital heart disease or chronic lung diseases, administered in five doses during the season [11,21,25]. To conclude, the vaccine covers the group of older adults over 60 years of age, since they are also a population at risk of respiratory syncytial virus.

## 20. Strengths, Limitations, and Knowledge Gaps in the Current Evidence

Current evidence supporting maternal RSV vaccination is largely based on randomized clinical trials, particularly the phase 3 MATISSE study, which provides robust efficacy and safety data derived from rigorous study methodology. Nevertheless, although randomized trials offer high internal validity, their findings may not be fully generalizable to routine clinical practice because trial participants are selected according to strict eligibility criteria and are managed under controlled conditions. In contrast, real-world studies provide valuable information regarding vaccine effectiveness in broader and more heterogeneous populations, reflecting everyday clinical settings, healthcare system variability, and implementation challenges. However, observational studies are inherently more susceptible to residual confounding, selection bias, and variations in healthcare access and reporting practices.

Despite the encouraging results observed in both clinical trials and real-world studies, several uncertainties remain. Most available effectiveness data originate from high- and middle-income countries, whereas evidence from low-income settings, where the burden of severe RSV disease is frequently greatest, remains scarce. Additionally, the duration of protection beyond the first six months of life has not been fully established, and there are currently insufficient data to support revaccination during subsequent pregnancies. Long-term safety surveillance and further studies evaluating maternal vaccination across different epidemiological contexts are still needed. Furthermore, given the rapidly evolving evidence base and regulatory landscape for maternal RSV immunization, recommendations regarding vaccination timing, implementation strategies, and preventive approaches may continue to change as additional clinical trial data, real-world evidence, and post-marketing surveillance findings become available. Therefore, healthcare professionals and policymakers should regularly consult updated guidance issued by national and international health authorities.

Finally, implementation challenges, including vaccine access, cost-effectiveness, seasonal administration strategies, and equitable integration into prenatal care programs, particularly in low-resource settings, represent important areas requiring further investigation. Addressing these evidence gaps will be essential for optimizing maternal RSV immunization strategies worldwide.

## 21. Future Priorities for Research and Monitoring

Looking ahead, multicenter studies with larger samples and greater population diversity are needed, encompassing different geographic regions and ethnic groups, to broaden the representativeness of the results. Additional clinical trials are also needed that evaluate in greater detail the safety and efficacy of vaccination against respiratory syncytial virus during pregnancy, especially in cases of prematurity. In addition, future research should investigate the concomitant administration of the RSV vaccine with other vaccines recommended in pregnancy, as well as the need for revaccination in subsequent pregnancies. The standardization of the outcomes analyzed and the obtainment of more robust data on gestational age at birth may contribute to a better understanding of the benefits of maternal immunization and to the improvement of clinical recommendations [29].

## 22. Conclusions

RSV remains one of the leading causes of severe lower respiratory tract infection, hospitalization, and mortality among young infants worldwide, particularly during the first months of life, when immunological and anatomical immaturity increase susceptibility to severe disease. In this context, maternal immunization has emerged as a highly effective preventive strategy capable of bridging this critical period of vulnerability through the transplacental transfer of protective antibodies. The currently available evidence demonstrates that the RSVpreF provides substantial protection against severe RSV-associated disease, significantly reducing lower respiratory tract infections, hospitalizations, and healthcare utilization during early infancy. Furthermore, the vaccine has shown a favorable safety profile, with adverse events generally being mild and transient and with no consistent evidence of major maternal or neonatal safety concerns.

The success of maternal vaccination is supported by its biological rationale, its integration into routine prenatal care, and its potential to generate meaningful public health benefits. Although continued surveillance remains important, particularly regarding prematurity and hypertensive disorders of pregnancy, current data indicate that the benefits of vaccination outweigh the potential risks. Maternal immunization should not be viewed as a replacement for passive immunization with long-acting monoclonal antibodies but rather as a complementary strategy within a broader RSV prevention framework, allowing for individualized protection according to maternal, neonatal, and epidemiological circumstances.

As RSV prevention strategies continue to evolve, expanding vaccine coverage, improving access to prenatal care, strengthening healthcare professional education, and maintaining robust post-marketing surveillance will be essential to maximize the impact of maternal immunization programs. Future advances may include the development of next-generation RSV vaccines with broader and longer-lasting protection, optimization of maternal immunization strategies, and the incorporation of pediatric vaccine platforms currently undergoing clinical evaluation. In addition, generating high-quality evidence from low- and middle-income countries and evaluating vaccination strategies in subsequent pregnancies remain important research priorities. Overall, maternal RSV vaccination represents one of the most important recent advances in perinatal infectious disease prevention and has the potential to substantially reduce the global burden of RSV-associated morbidity and mortality in early childhood.

## Figures and Tables

**Table 1:** Diagnostic methods for respiratory syncytial virus infection.

Method	Turnaround Time	Sensitivity	Advantages	Limitations
Viral culture	3–6 days	High	Viral characterization	Slow
Antigen detection (DFA/EIA ^1^)	Minutes	Moderate	Rapid, inexpensive	Lower sensitivity
RT-PCR ^2^	Hours	Highest	Gold standard	Requires specialized laboratory

^1^ DFA/EIA. Direct Fluorescent Antibody assay/EIA. Enzyme Immunoassay. ^2^ RT-PCT. Reverse Transcription–Polymerase Chain Reaction.

**Table 2:** Regulatory recommendations for maternal respiratory syncytial virus vaccination.

Organization	Recommendation	Gestational Age	Key Considerations
FDA ^1^	Recommended	32–36 weeks	Precautionary window due to preterm birth signal
CDC/ACIP ^2^	Recommended	32–36 weeks	Seasonal administration
ACOG ^3^	Recommended	32–36 weeks	Can be coadministered with Tdap, influenza, and COVID-19 vaccines
EBCOG ^4^	Recommended	According to national policies	Regional adaptation according to RSV epidemiology
ANVISA ^5^	Approved	24–36 weeks	Approved for maternal immunization
Brazilian Ministry of Health	Incorporated into public health strategies	National implementation	Infant protection during early life

^1^ FDA—Food and Drug Administration. ^2^ CDC/ACIP—Centers for Disease Control and Prevention/Advisory Committee on Immunization Practices. ^3^ ACOG—American College of Obstetricians and Gynecologists. ^4^ EBCOG—European Board and College of Obstetrics and Gynaecology. ^5^ ANVISA—Brazilian Health Regulatory Agency (Agência Nacional de Vigilância Sanitária).

**Table 3:** Vaccines and monoclonal antibodies for respiratory syncytial virus prevention.

Strategy	Target Population	Mechanism	Duration of Protection	Main Limitation
Maternal RSVpreF vaccine ^1^	Pregnant women	Transplacental antibodies	First 6 months of life	Requires adequate timing before delivery
Nirsevimab	Infants	Passive immunization	Entire RSV ^2^ season	Cost and access
Palivizumab	High-risk infants	Passive immunization	Monthly doses during season	Multiple administrations

^1^ RSVpreF vaccine = Respiratory Syncytial Virus Prefusion F Protein Vaccine. ^2^ RSV. Respiratory syncytial virus.

**Table 4:** Key Clinical recommendations for maternal respiratory syncytial virus immunization.

Clinical Aspect	Recommendation	Comments/Clinical Considerations
Target population	Pregnant women eligible according to national recommendations	Maternal vaccination should be offered to pregnant women within the approved gestational window and according to local regulatory guidance.
Recommended vaccine	RSV prefusion F protein vaccine (RSVpreF vaccine; Abrysvo^®^)	Currently, the only RSV vaccine approved for maternal immunization in several countries.
Gestational age for vaccination	Administer according to national recommendations	FDA, CDC/ACIP, and ACOG recommend vaccination between 32 and 36 weeks of gestation, whereas ANVISA approval includes administration between 24 and 36 weeks of gestation.
Optimal timing	Vaccinate sufficiently before delivery	Administration at least 14 days before birth is desirable to maximize transplacental antibody transfer and neonatal protection.
Expected neonatal benefits	Reduction in severe RSV-associated disease	Maternal vaccination reduces severe RSV-associated lower respiratory tract infection, hospitalization, oxygen supplementation, and intensive care admission during early infancy.
Mechanism of protection	Passive transfer of maternal antibodies	Vaccine-induced maternal IgG antibodies are actively transferred across the placenta via FcRn receptors, providing protection from birth.
Coadministration with other maternal vaccines	Generally acceptable	Maternal RSV vaccine may be administered concomitantly with other recommended maternal vaccines, including influenza, Tdap, and COVID-19 vaccines, according to local recommendations.
Contraindications	Follow product labeling and national guidance	Contraindications include a history of severe allergic reaction to a previous dose or to any vaccine component.
Preterm birth considerations	No causal association established	Although numerical imbalances in preterm birth were observed in clinical trials, no statistically significant increase or causal relationship has been demonstrated.
Hypertensive disorders of pregnancy	Continue routine obstetric surveillance	Slight numerical differences were observed in clinical trials; however, no causal association has been established.
Use of nirsevimab	Consider in specific situations	Particularly relevant for infants born to unvaccinated mothers, infants born <14 days after maternal vaccination, preterm infants, or when maternal vaccination is contraindicated.
Maternal vaccination versus nirsevimab	Complementary strategies	Maternal vaccination and nirsevimab should be viewed as complementary rather than competing approaches for RSV prevention.
Implementation considerations	Integrate into routine prenatal care	Successful implementation depends on healthcare provider recommendation, vaccine acceptance, equitable access, and robust public health policies.
Post-marketing surveillance	Continue long-term monitoring	Ongoing surveillance is essential to assess long-term effectiveness, rare adverse events, duration of protection, and vaccine impact on RSV epidemiology.

Abbreviations: RSV, respiratory syncytial virus; RSVpreF, respiratory syncytial virus prefusion F protein vaccine; FDA, Food and Drug Administration; CDC, Centers for Disease Control and Prevention; ACIP, Advisory Committee on Immunization Practices; ACOG, American College of Obstetricians and Gynecologists; ANVISA, Brazilian Health Regulatory Agency; Tdap, tetanus, diphtheria, and acellular pertussis vaccine; IgG, immunoglobulin G; FcRn, neonatal Fc receptor.

## Data Availability

The data presented in this study are available on request from the corresponding author.
